# Authors’ reply

**Published:** 2011

**Authors:** Anish P Nagpal, Harshad Soni, Sanjiv Haribhakti

**Affiliations:** Department of Surgical Gastroenterology, Haribhakti Surgical Hospital, Ahmedabad, India

Dear Sir,

We appreciate your keen interest in our article[1] and your comments[2] are well taken. There is no doubt that 24-hour ambulatory pH metry and manometry are helpful before Laparoscopic Antireflux surgery, and in the western literature, they are considered to be the gold standard before surgery for selection of a particular procedure, either a total or a partial fundoplication. Few patients with abnormal oesophageal peristalsis will certainly not do well after a complete 360 degree Fundoplication. However, pH metry and manometry are not widely available even in major cities, in developing countries like India. We did this retrospective study to look at our single surgeon experience of Laparoscopic Antireflux Surgery, done without pH metry and manometry. We routinely perform the complete 360 degree Floppy Nissen’s Fundoplication procedure for all patients undergoing antireflux surgery. In our study of 46 patients, only one patient (2.18%) required conversion from 360 degree Fundoplication to Partial Toupet Fundoplication. Certainly this patient would have benefitted if all the 46 patients would have had a preoperative pH metry and manometry. However, in our experience 98% of the patients have a satisfactory outcome with the 360 degree Fundoplication done without pH metry and manometry. These results match with the results of other studies,[3] suggesting a similar outcome after a routine preoperative pH metry and manometry.
